# Nutritional Quality during Development Alters Insulin-Like Peptides’ Expression and Physiology of the Adult Yellow Fever Mosquito, *Aedes aegypti*

**DOI:** 10.3390/insects9030110

**Published:** 2018-08-30

**Authors:** Rana Pooraiiouby, Arvind Sharma, Joshua Beard, Jeremiah Reyes, Andrew Nuss, Monika Gulia-Nuss

**Affiliations:** 1 Department of Agriculture, Nutrition, and Veterinary Sciences, University of Nevada, Reno, NV 89557, USA; pooraiiouby@googlemail.com; 2 Department of Biochemistry and Molecular Biology, University of Nevada, Reno, NV 89557, USA; arvinds@unr.edu (A.S.); jdbeard93@gmail.com (J.B.); jeremiahbreyes@nevada.unr.edu (J.R.)

**Keywords:** insulin-signaling, insulin receptor, *Aedes aegypti*, mosquitoes, insulin, insulin-like peptides, nutrition, hyperinsulinemia, larval-diet, metabolic reserves

## Abstract

Mosquitoes have distinct developmental and adult life history, and the vectorial capacity of females has been shown to be affected by the larval nutritional environment. However, little is known about the effect of developmental nutrition on insulin-signaling and nutrient storage. In this study, we used *Aedes aegypti*, the yellow fever mosquito, to determine whether larval nutrition affects insulin gene expression. We also determined the traits regulated by insulin signaling, such as stored-nutrient levels and fecundity. We raised mosquito larvae on two different diets, containing either high protein or high carbohydrates. Development on a high-carbohydrate diet resulted in several life-history phenotypes indicative of suboptimal conditions, including increased developmental time and decreased fecundity. Additionally, our data showed that insulin transcript levels are affected by a high-carbohydrate diet during development. Females, not males, reared on high-carbohydrate diets had much higher transcript levels of insulin-like peptide 3 (ILP3), a mosquito equivalent of human insulin, and these females more readily converted sugar meals into lipids. We also found that AaILP4, not AaILP3, transcript levels were much higher in the males after a sugar meal, suggesting sex-specific differences in the insulin-signaling pathway. Our findings suggest a conserved mechanism of carbohydrate-mediated hyperinsulinemia in animals.

## 1. Introduction

A variety of environmental factors such as light, temperature, and nutrient availability affect the development and physiology of animals. Contending with changes in nutrition often involves hormonal regulation and metabolic homeostasis [1]. In mammals, insulin and glucagon are key systemic regulators which maintain circulating glucose levels during fluctuating nutritional conditions [2]. Similarly, hemolymph carbohydrate levels in insects are regulated by the action of two endocrine hormone families: insulin-like peptides (ILPs), which are structurally and functionally analogous to vertebrate insulin, and adipokinetic hormone, a functional analog of vertebrate glucagon [3,4,5].

Insulin/Insulin-like growth factor signaling in the yellow fever mosquito, *Aedes aegypti*, is mediated by eight ILPs (AaILPs). AaILP1, AaILP3, and AaILP8 are predominantly expressed in the adult brain, AaILP4 and AaILP7 are expressed in both brain and ovaries, and AaILP2, AaILP5, and AaILP6 are ubiquitously expressed in most adult tissues [6]. AaILP3 has been shown to bind to and signal through a tyrosine kinase receptor, the mosquito insulin/Insulin growth factor receptor (MIR), and it is predicted that more than one ILP is capable of binding to this common MIR. AaILP3 modulates circulating carbohydrate, glycogen, and lipid levels [7,8]. AaILPs serve additional roles in coordinating blood meal digestion and yolk deposition in adult female *Ae. aegypti* [9]. Ecdysteroid synthesis by ovaries requires five times higher AaILP4 levels compared to AaILP3, and unlike AaILP3, it does not affect circulating carbohydrates, stored glycogen, and lipid levels [10]. Additionally, AaILP3 in *Ae. aegypti*, but not in *Ae. atropalpus*, regulated ecdysteroid synthesis in ovaries and yolk synthesis in eggs [11]. However, native AsILP3 isolated from *Anopheles stephensi*, was capable of stimulating ecdysteroid synthesis in diverse mosquito lineages including *An. gambiae, An. stephensi, Ae. Aegypti*, and *Culex quinquefasciatus*, suggesting its functional role in reproduction is generally conserved in mosquitoes, despite the exception noted in *Ae. atropalpus* (Nuss and Brown, 2018). AaILP7 and AaILP8 have recently been shown to regulate glycogen and lipid metabolism and ovarian development [12]. AaILP7 modulates lipid deposition before a blood meal for egg development, and AaILP8 appears to modulate lipid mobilization. Glycogen levels exhibited opposite trends in these mosquitoes, which indicate that AaILP7 and AaLP8 are also involved in the regulation of sugar storage [12]. Functions of the other four ILPs in *Ae. aegypti* have not yet been explored. 

Nutrient quantity and quality affect the expression of ILPs in other insects. For instance, in the honey bee, *Apis mellifera,* which has two ILP genes, *AmILP1* and *AmILP2*, *AmILP1* was upregulated when fed on a high protein diet [13]. Most species of mosquito larvae are non-selective filter feeders of organic particles suspended in water and of micro-organisms such as bacteria, viruses, protozoans, and fungi [14]. Larval instars 3 (L3) and L4 feed vigorously and store nutrients for the non-feeding pupal stage; therefore, information on nutrient signaling is critical during this developmental period. Given the complex compensatory expression of ILPs, we hypothesized that the ILPs will have different expression profiles in *Ae. aegypti* and would change in response to the quality and composition of the larval diet. To test this hypothesis, we chose two commonly used commercial diets for mosquito rearing that differ in carbohydrate:protein composition and measured the effects of these larval diets on ILP expression. We also studied the impacts on subsequent adults eclosed from the larvae fed on these diets. Thirdly, we measured levels of circulating carbohydrates, stored glycogen, lipids, and proteins, as well as life history traits such as developmental time, body size, lifespan, and fecundity. Our data suggest that diets consisting of different protein:carbohydrate ratios result in differential expression of AaILPs. The high protein diet resulted in shorter developmental time, higher lipid levels, and more fecund females compared to the high carbohydrate diet. There was no significant difference in body size in males whereas females that had consumed protein-rich diet were significantly bigger than those that had fed on the carbohydrate-rich diet. Adults eclosed from the protein-rich diet had a longer lifespan. Our results also suggest differences in nutrient metabolism between males and females: females with a diet low in protein (high in carbohydrates) during development tend to convert the sugar meal into lipid more readily than males. Developmental nutrients did not have any significant effect on stored metabolites in males, suggesting that females require higher lipid stores, presumably for host-seeking and reproduction.

## 2. Materials and Methods

### 2.1. Mosquitoes

The UGAL strain of *Ae. aegypti* was used in all experiments. All stages were maintained in an insectary under a 16 h light: 8 h dark photoperiod and a temperature of 27 °C and 80% relative humidity [11]. Eggs were hatched overnight in deionized water. Larvae were reared in plastic containers. One hundred and fifty first instar larvae were counted and reared in 500 mL de-ionized water. Equal amounts (by weight) of standard fish food (Tetramin^®^, Melle, Germany) or gerbil food (Small World^®^, St. Louis, MO, USA) were ground to fine powder and were provided to larvae every day. These meridic diets as such are not tightly formulated, yet are commonly used in mosquito labs around the world and provide an accessible diet for comparisons of bulk nutrient proportions. We determined that the fish food diet (protein rich, hereafter referred to as PR) contained 50% protein, 15% carbohydrates, and the gerbil food diet (carbohydrate rich, hereafter referred to as CR) contained approximately 6% protein, 83% carbohydrate (Figure S1). These proportions differed from those reported on the product labels, but contained similar imbalances in carbohydrate:protein ratio. 

The standard larval diet was as follows: days 1 and 2 post larval emergence—150 mg diet/pan, days 3 and 4—300 mg/pan, day 5 and 6—600 mg/pan, day 7 onward—no diet. Most mosquitoes on the PR diet pupated by day 7–8, and most on the CR diet pupated by day 10. Pupae were collected from the rearing pans and kept in adult cages for emergence. Adults had access to water and a 10% sucrose solution ad libitum unless otherwise stated. Females were blood fed on day 4 post eclosion. 

### 2.2. Quantitative Expression Analysis of ILPs by Real Time RT-PCR

The quantitative expression profiles of AaILPs were conducted in fourth instar larvae and adult male and female mosquitos reared on PR and CR food regimes. Five L4 were pooled for RNA extraction. Adult mosquitoes (10 mosquitoes per sample) were collected for RNA isolation at two time points: (1) 24 h post eclosion without providing any sugar, and (2) 24 h post eclosion fed on 10% sugar solution for 10 min, with samples collected one hour post sugar meal. Total RNA was isolated from a pool of five larvae per replicate (three replicates per cohort) and ten adults per replicate (three replicates per time point per cohort) using TRIzol reagent according to manufacturer protocols (Invitrogen, Waltham, MA, USA). Total RNA quantity was measured with Nanodrop spectrophotometer. Five µg of total RNA was used for DNase treatment (Sigma, St. Louis, MO, USA) according to the manufacturer’s protocol. DNase-treated RNA samples were re-purified with Trizol. Purity was determined by 260/280 and 260/230 ratios, with acceptable values in the ~2.0 and 2.0–2.2 range, respectively (all samples collected met purity standards). One µg of DNase treated RNA was used for cDNA synthesis with iScript reverse transcription supermix (BioRad, Hercules, CA, USA). cDNA was diluted 10x in water before using it as a template in qRT-PCR experiments. One µL cDNA was used in each 10 µL qRT-PCR reaction. Each sample was run in triplicate wells of 96-well plate. qRT-PCR was performed on a CFX touch Real-Time PCR Detection system (BioRad, Hercules, CA, USA), using SYBR green master mix (BioRad). Sequences of specific primers for AaILPs, MIR, and a housekeeping gene, ribosomal protein s7, are listed (Table 1). All reactions were performed with 120 s at 95 °C, followed by 40 cycles of 10 s at 95 °C, 15 s at 58 °C, and 15 s at 72 °C, and melt curve was analyzed at 70–95 °C. Relative expression was calculated using the 2^−ΔΔCt^ method, where PR diet fed mosquitoes were considered control and expression of ILPs and MIR in CR diet fed mosquitoes was calculated relative to the control levels. Experiments were replicated with four different mosquito cohorts.

### 2.3. Mosquito Physiology Bioassays

#### 2.3.1. Developmental Time

The number of larvae molted to L2, L3, L4, and pupal stages were counted daily. Exuviae and dead larvae were removed each day from the pans. Pupae were collected and transferred to emergence cages. This experiment was repeated for a total of five different cohorts.

#### 2.3.2. Fecundity

Five females per replicate per treatment were kept individually in small cages made out of specimen cups, lined with a moist paper towel. The number of eggs deposited by each female was counted 5 days post blood meal (PBM). Females that did not deposit eggs were dissected to examine the ovaries for developed oocytes. As above, this experiment was repeated on five different mosquito cohorts.

#### 2.3.3. Metabolic Assays

Trehalose, glycogen, lipid, and protein levels were quantified in mosquitoes (1) 24 h post eclosion, that had been provided with deionized water only without a sugar meal (teneral reserves), and (2) 20 h post eclosion, fed on 10% sucrose solution for 4 h, and 20 h post sugar meal (allocation of nutrients from a sugar meal for storage). Sugar uptake was confirmed by looking at the mosquitoes under the microscope for swollen abdomens and blue color from food dye in the sugar solution, and unfed mosquitoes were discarded. Samples were processed from both males and females to separate micro-metabolites. In brief, two adults per sample were placed in tubes containing 100 µL of Na_2_SO_4_ and 200 µL of methanol where they were either homogenized or stored at −80° C. These samples were further processed into phases containing trehalose (circulating carbohydrates), glycogen, and lipids as previously described [7,8]. Levels of trehalose and glycogen were estimated with the Anthrone assay and lipids with the Vanillin assay [8]. For the protein assay, two adults per sample were collected in phosphate buffer saline with protease inhibitor (PBS+ PI) (Complete Mini; Roche Applied Science, Indianapolis, IN, USA). Total proteins were estimated with Bradford assay. 

### 2.4. Data Analysis

All data were analyzed using the GraphPad Prism 7 software (La Jolla, CA, USA). AaILP data was analyzed with an unpaired *t*-test between control (PR) and experimental (CR) groups. Mosquito teneral reserves were examined using a one-way ANOVA followed by the Tukey’s multiple comparisons test with each treatment serving as the independent variable. The effect of diet on the number of eggs deposited was examined using a *t*-test. 

## 3. Results 

### 3.1. ILP Expression Changes in Mosquitoes Reared on Different Diets 

**Figure 1:**
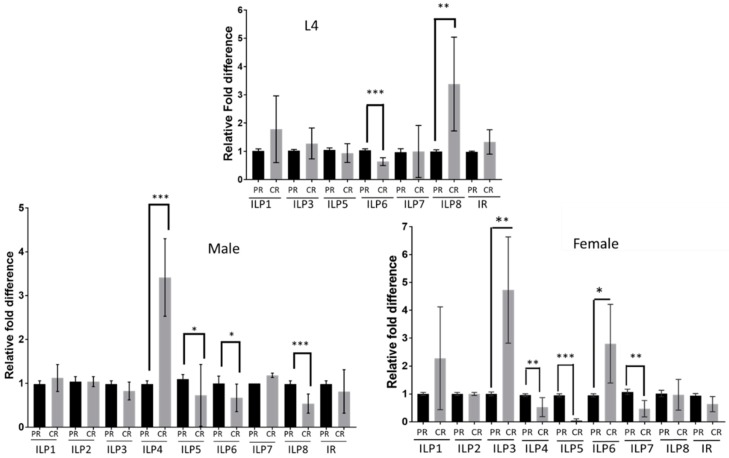
Transcript levels of insulin-like peptides in L4 and adult mosquitoes from larvae reared on different diets. ILPs transcript levels were determined by qRT-PCR in larval instar four (L4, top) male (left) and female (right) adults eclosed from the larvae reared either on PR or CR diet. PR diet fed samples were used as a control for the relative transcript expression determination. Significant differences in transcript abundance were determined with an unpaired *t*-test (* *p* < 0.05; ** *p* < 0.01; *** *p* < 0.0001). Experiments used four different cohorts and each cohort consisted of three replicates (*n* = 12). ILP2 and ILP4 expression was not detected in L4s. Mean ± SD were plotted. PR = protein-rich diet; CR = Carbohydrate-rich diet.

*Ae. aegypti* larvae hatched from the same egg cohort were fed on equal amounts (by weight) of either PR or CR diet from larval emergence to pupal molt. Relative ILP transcript expression was normalized to PR diet-fed mosquitoes for each group. In L4 larvae, AaILP8 was highly expressed, whereas AaILP 6 expression was low (Figure 1); AaILP7 expression was variable and was not statistically different. In adult males, AaILP4 was upregulated, whereas it was downregulated in females. Similarly, AaILP6 showed a reversed trend; expression was significantly higher in females and lower in males. AaILP3 transcripts were significantly higher in females. AaILP5 expression was low in both adult males and females, whereas AaILP7 and AaILP8 were less expressed only in females and males, respectively (Figure 1). AaILP4 was expressed in adults but not in the larval stages in either food group. AaILP2 was barely detectable in any of the life stages tested (Ct values over 38), and no changes in expression were detected.

### 3.2. Protein Rich Diet Resulted in Shorter Developmental Time

Larvae fed on a PR diet completed their development to pupation in 7–8 days, which is standard in our laboratory and others [15]. However, the CR diet fed larvae delayed pupal emergence by 2 days. Developmental delay was detected as early as the L2-L3 molt (Table 2). The effect on developmental time was likely not due to avoidance of food because we observed similar amounts of ground food at the bottom of the larval containers across treatments (personal observation).

### 3.3. Levels of Circulating Carbohydrates, Glycogen, and Lipids Varied in Adults from Different Diet Fed Larvae, whereas Protein Levels Remained Constant

**Figure 2:**
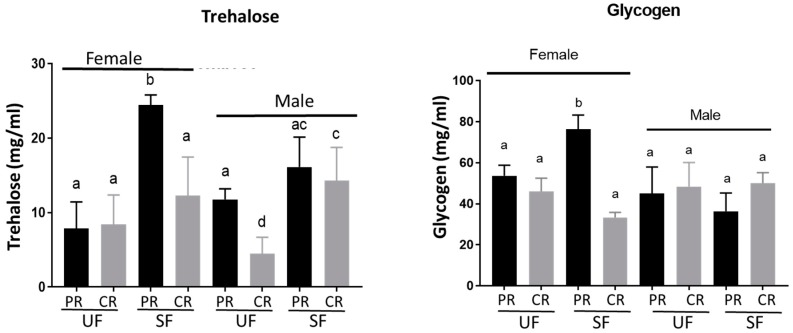

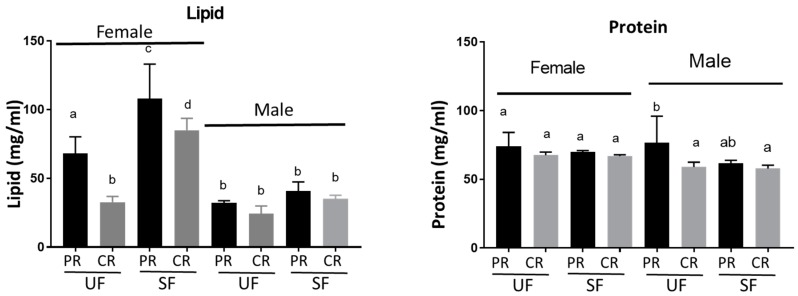
Effect of larval diets on adult trehalose, glycogen, lipid, and protein levels. Two 24 h old, unfed adult female and male mosquitoes per cohort from PR and CR groups were used to assess teneral nutrient reserves. Additional mosquitoes from the same cohort were also sugar-fed for 4 h and then processed 20 h later to assess differences in nutrient storage. Levels of glycogen, trehalose, lipids, and proteins were determined in females (first four vertical bars) and males (last four vertical bars) reared on PR (black bars) or CR (gray bars) diets (mean ± SD). One-way ANOVA followed by Tukey’s multiple comparison was used. Glycogen: F (D_Fn_, D_Fd_): F (7, 40) = 22.45, *p* < 0.0001; Trehalose: F (7, 64) = 26.89, *p* < 0.0001; Lipids: F (7, 40) = 77.68, *p* < 0.0001. Protein: F (7, 40) = 4.419, *p* = 0.0010. Experiments were performed on four samples from each of three different cohorts. PR = protein-rich diet fed; CR = carbohydrate-rich diet fed; UF = unfed; SF = sugar-fed.

Levels of circulating carbohydrates (trehalose) and stored nutrients (glycogen, lipids, and protein) were measured in adult females and males at 24 h (before sugar meal) and 48 h (after a sugar meal) post-eclosion. In newly eclosed, unfed males, levels of glycogen and lipid were equal in both PR and CR diet fed animals; however, trehalose and protein levels were significantly lower in males reared on CR diet compared to those fed on a PR diet (Figure 2). In sugar-fed males, levels of all metabolites were equal in both diets. In females, the effect of larval diets was more pronounced. In newly eclosed, unfed females, lipid levels were significantly lower in the CR diet group, whereas glycogen, trehalose, and protein levels were equal in both diets (Figure 2). After a sugar meal, levels of glycogen, trehalose, and lipids increased significantly in sugar-fed females raised on the PR diet compared to the unfed control, whereas protein levels did not change (Figure 2). In the CR diet samples, the sugar meal had no significant effect on the levels of glycogen, trehalose, and protein, whereas lipid levels increased significantly compared to unfed females. Except for protein, metabolite levels were significantly higher in the PR diet females compared to the CR diet females after a sugar meal (Figure 2).

### 3.4. Protein-Rich Diet Resulted in Higher Fecundity

**Figure 3:**
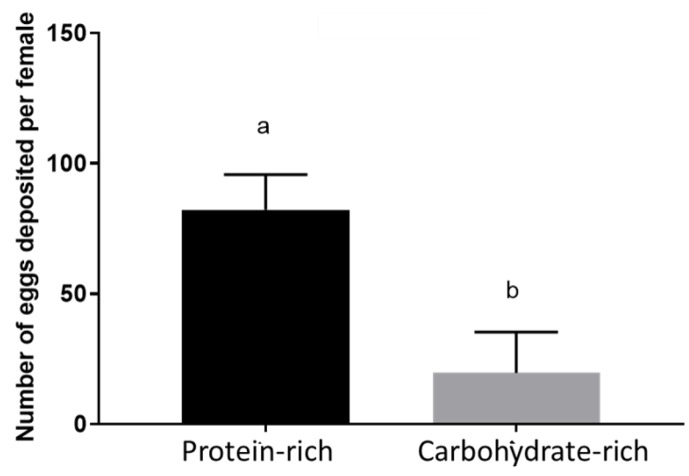
Effect of larval diets on fecundity. A subset of five females per treatment were kept individually in small containers lined with moist paper towels to provide an oviposition surface. The number of eggs deposited by individual females was counted five days post-blood meal. Mean ± standard error were plotted. Data were analyzed by *t*-test (*t* = 9.599, df = 18), *p* < 0.0001. Experiments were replicated thrice with different cohorts. N = 15.

Females that consumed a CR diet as larvae were less fecund. The PR diet group deposited on average 82 eggs, whereas the CR diet group deposited only 20 eggs on average (Figure 3). 

### 3.5. Protein-Rich Diet Resulted in Longer Life Span and Difference in Female Body Size

Females eclosed from a CR diet were more susceptible to dying earlier than males. Thirty percent of males on the PR diet survived longer than those on the CR diet, whereas this difference was 50% in females (Figure S2). There was no difference in body size in males, whereas females reared on the PR diet were significantly larger than those reared on the CR diet (Figure S3).

## 4. Discussion

Our data suggest that subsisting on a carbohydrate-rich diet during larval development alters mosquito insulin-like peptide expression and affects nutrient storage, larval development time, and fecundity. Nutrition is a primary determinant of lifespan and reproductive capacity in all organisms, from yeast to humans [16,17,18], and its relationship to life history has been studied extensively. Larval nutrition can have far-reaching effects on adult traits [19,20,21,22,23]. A wide array of insect literature has found that calorically deficient food during development leads to increased developmental time, decreased adult weight [23,24,25,26], and reduced fecundity. However, the effect of nutrient quality and the relationship with ILPs that maintain nutrient homeostasis has not yet gained much attention. 

*Ae. aegypti* ILPs 1–8 have been identified and named mostly through their sequence homology to the *Anopheles gambiae ILPs* [6] and the canonical B-C-A domain structure as observed in mammalian insulin [4]. Similar to *Ae. aegypti* ILPs1-8 (AaILPs), eight ILPs have been identified in *Drosophila melanogaster* genome. Dilp8 was recently identified based on the predicted arrangement of conserved cysteines [27,28]. Outside of these conserved cysteines, most members of insulin superfamily in Diptera only share a few key conserved amino acids, and except for dilp7, that has high amino acid sequence similarity to AaILP5; the AaILPs do not have high sequence similarity to dilps [6] and therefore sequence homology provides marginal guidance to their function between these different organisms.

Our data here suggest that manipulating food quality during larval development has significant effects on AaILPs expression. In *D. melanogaster*, starvation reduced larval dilp3 and dilp5 expression [29], whereas starvation increased dilp6 expression in larvae and adults [30,31]. When maintained on yeast-restricted or diluted diets, dilp5 expression was downregulated in both larval and adult flies [32,33,34]. We observed lower expression of AaILP6 in the CR diet fed larvae; AaILP7 was not statistically different from the protein-rich diet fed larvae, however, its expression was variable. AaILP8 was the most significantly upregulated ILP in L4s. In *D. melanogaster*, Dilp8 transcript levels peak during the transition between larval instars and have been shown to control the timing of larval molts, ensuring that animals do not progress to the next developmental stage before adequate growth has occurred [28]. Higher transcript levels of AaILP8 in the L4 might have a similar function in regulating the molt to the pupal stage. The difference in AaILP8 peak in CR vs. PR diet may be due to the difference in the timing of L4s since the CR diet fed mosquitoes took two to three extra days to become L4, and the samples were collected on different days. However, no functional or structural evidence suggest dilp8 and AaILP8 are homologs. Previous attempts to elucidate the function of AaILP8 by injecting adult mosquitoes with synthetic versions of this peptide had no effect on adult nutrient metabolism (unpublished data, Gulia-Nuss), in contrast to synthetic AaILP3 [9]. However, a recent study suggested a role of AaILP8 in lipid mobilization after a blood meal [12]. 

Feeding *D. melanogaster* adults a diet comprised of different protein-to-carbohydrate ratios at variable caloric concentrations resulted in higher *dilp5* transcript level with increased protein levels [35]. Our data also showed higher AaILP7 expression in PR diet fed females. AaILP5 transcript levels were higher in protein rich fed adults of both sexes, suggesting a functional similarity with dilp5. AaILP3 has been shown to be the mosquito equivalent of mammalian insulin in both structure and function [7]. A single injection of synthetic ILP3 in sugar-fed, decapitated *Ae. aegypti* females was able to restore trehalose, glycogen, and lipid levels similar to the head-intact control [7]. Our data supports these previous findings and further strengthens the role of AaILP3 in adult female nutrient metabolism. The function of ILP5 has not been studied in *Ae. aegypti* and is worth further exploration. 

AaILP4 does not bind directly to the MIR and had no effect on nutrient metabolism in sugar-fed *Ae. aegypti* females [10]. AaILP4 was expressed in both male and female adults but not in the larval stages in either food group. Expression of AaILP4 in males is an interesting finding because this ILP was previously considered female-specific [10]. The higher expression of AaILP4 in recently-eclosed males fed on the CR diet might shed light on the possible functions of this ILP. *Ae. aegypti* ILP6 has been suggested to be structurally similar to mammalian insulin-like growth factors because of a shorter C chain [6]. Further investigation of potential growth-factor-like functions is needed, as is the role of increased expression of this ILP in CR diet fed females. Unlike *D. melanogaster*, MIR expression was constant in all mosquito life stages, suggesting regulation at the ligand (ILP) level.

Our data show that different diets during larval development has significant effects on adult mosquito physiology. Mosquitoes reared on equal amounts (*w*/*w*) of PR food lived longer (Supplementary Figure S1) and deposited more eggs compared to those reared on CR diets. The effect of different larval diets was also apparent in levels of stored and circulating nutrients in adults. Male mosquitoes eclosed from the CR diet had lower levels of trehalose; however, glycogen, lipid, and protein levels were equal. 

Energetic needs change dramatically between life stages in holometabolous insects, particularly in female insects, since nutrient investment into eggs constitutes a major expenditure of energy [36]. Therefore, most studies have focused on female nutrient allocation. The nutritional environment experienced by insect larvae strongly influences female fitness-related traits such as body size, teneral metabolic reserves, and fecundity [37,38,39]. In *A. aegypti*, approximately 80% of lipids found in eggs are derived from regular sugar meals before blood feeding [40]. Because egg development is dependent on a female’s reserve of nutrients, it is important that she restrict oogenesis until sufficient nutrients are available, otherwise she risks starvation. Presumably the endocrine and nervous systems monitor these reserves and regulate physiological, developmental, and behavioral processes that rely on these reserves accordingly. In our study, there was no difference in body size in males whereas females reared on the PR diet were significantly bigger than CR diet (Supplementary Figure S2). Adults eclosed from the PR diet fed larvae had longer lifespan. Both males and females that consumed the CR diet died sooner than mosquitoes that had been exposed to a PR diet, but this trend was more pronounced for females (30% increased survivorship in males vs. 50% in females) (Supplementary Figure S2). In *Anopheles stephensi*, larvae reared under a reduced diet also had increased adult mortality [41]. Two recent studies in *D. melanogaster* suggested different results of the influence of larval diet on adult lifespan. Davies et al. [42] suggested a positive correlation between adult diet and lifespan increase; however, there was no effect of larval diet on adult lifespan and it was concluded that fitness consequences due to developmental environment can be ‘cancelled out’ during adulthood. However, another study [43] concluded that flies fed a high carbohydrate diet during development displayed a bimodal distribution of lifespans composed of a short-lived population and a long-lived population. These studies along with previous work and our own data suggest a more complicated regulation of lifespan than a simplistic view of caloric intake. 

Our results also suggest differences in nutrient metabolism between males and females. When fed CR food during development, adult females, but not males, converted the adult sugar meal into lipid reserves more readily. It is also notable that the CR reared females had lower lipid levels at eclosion, and this increase in lipids post sugar meal might be required for maintenance of homeostasis. In *Ae. sollicitans*, when the adults were maintained on sugar meal only, females had higher levels of lipid than males [44]. When females were starved until no lipid remained and then fed on sugar, lipid levels increased, and newly synthesized lipid appeared, entirely made from sugars [45], further suggesting that optimum lipid levels are needed for females to survive. In mammals, high carbohydrate and low protein intake is positively associated with hyperlipidemia. Low-carbohydrate, high-protein, low-energy diets are widely used in weight management programs. A growing body of evidence suggests that high-protein, low-carbohydrate diets improve hyperinsulinemia [46]. These results suggest a similar control mechanism of metabolic stores and insulin signaling in both vertebrates and invertebrates. Analyzing how nutrient composition associates with the expression of ILPs reveals that nutritional status is modulated by different combinations of ILPs in males and females. Formulation of holidic larval diets may permit a more precise examination of ILP modulation in future studies. Further, our explorations with CRISPR-Cas9 knockouts will permit deeper examination of the roles of each specific ILP in mosquito nutrient homeostasis.

## 5. Conclusions

Our results suggest that diets consisting of different protein: carbohydrate ratios result in differential expression of ILPs. The high protein diet resulted in shorter developmental time, higher lipid levels, and more fecund females compared to the high carbohydrate diet. Adults eclosed from the protein-rich diet had a longer lifespan. Our results also suggest differences in nutrient metabolism between males and females: developmental nutrients did not have any significant effect on stored metabolites in males, suggesting that females require higher lipid stores, presumably for host-seeking and reproduction. Together, these observations suggest that different developmental diets used for rearing mosquitoes can influence the insulin-signaling mediated life-history traits during adult life, and these affects vary in males and females, suggesting sex-specific differences in nutrient regulation. 

## Figures and Tables

**Table 1:** Primer sequences for real-time RT-PCR. Sequences for all insulin-like peptides (ILPs), insulin receptor, and the housekeeping gene, ribosomal protein S7, are listed below.

Primer Name	Primer Sequence
ILP1 Fwd	5’-ACTGGTTTGCAACAGCTACC-3’
ILP1 Rev	5’-TCCAGGTCCTGTTTGATCTC-3’
ILP2 Fwd	5’-CATCACCGCTCAGAATACCT-3’
ILP2 Rev	5’-AGAACGGAAAACCGTGACTA-3’
ILP3 Fwd	5’-ACCAACTTGCGAGTATCGAG-3’
ILP3 Rev	5’-TGTACTACGGTTCCGACCAT-3’
ILP4 Fwd	5’-TACTCGAAGCACGACCCTAT-3’
ILP4 Rev	5’-GGCAACATTCCTCTACGATG-3’
ILP5 Fwd	5’-CTAATCCGGCACCTTTACTG-3’
ILP5 Rev	5’-AAGGGTAGCGCATTAGCAC-3’
ILP6 Fwd	5’-GAGCAAATCCACAACTCCAG-3’
ILP6 Rev	5’-GCACAGTTCCAAATTCCATC-3’
ILP7 Fwd	5’- GCGCCAACTATGACAAAACT-3’
ILP7 Rev	5’- AGGGTTTGTAGCAACAGTCG-3’
ILP8 Fwd	5’- AGGGCCATTCTACAAGCTCT-3’
ILP8 Rev	5’- AGGAATGTTTCTCCGTGTCC-3’
Insulin receptor Fwd	5’- AATGGTTACCGCCACTGAAG-3’
Insulin receptor Rev	5’- GCACTGATCCGCAGTACAGA-3’
Ribosomal protein S7 Fwd	5’- ACCGCCGTCTACGATGCCA-3’
Ribosomal protein S7 Rev	5’- ATGGTGGTCTGCTGGTTCTT-3’

**Table 2:** Means (hr ± SD) of CR larval diet developmental time compared to larvae reared on PR diet. Larvae were hatched from the same batch of eggs and reared under same conditions. Both sets were given equal amounts (by weight) of food (*n* = 6 cohorts of 100 larvae).

Diet	Developmental Time per Life Stage				Total
	L1 to L2	L2 to L3	L3 to L4	L4 to pupae	
PR	38 ± 5	44 ± 4	48 ± 5	48 ± 4	178 ± 18
CR	38 ± 4	50 ± 6	64 ± 6	84 ± 8	236 ± 24

## Supplementary Materials

The following are available online at http://www.mdpi.com/2075-4450/9/3/110/s1, Figure S1: Levels of proteins and carbohydrates in different diets. 150 mg each of protein-rich (PR) and carbohydrate rich (CR) diets were used to determine proportions of these macronutrients; Figure S2: Effect of larval diets on adult mosquito survival; Figure S3: Effect of larval diets on adult mosquito body size.

## Abbreviations

ILP	insulin-like peptides
Dilp	Drosophila insulin-like peptides
IR	insulin receptor
L1-4	Larval instars 1-4
RT-PCR	Reverse transcription Polymerase Chain Reaction
qRT-PCR	Reverse transcription Polymerase Chain Reaction
