# Supplementary material for: Nutritional Quality during Development Alters Insulin-Like Peptides’ Expression and Physiology of the Adult Yellow Fever Mosquito, Aedes aegypti
Source: Insects. 2018 Aug 30;9(3):110. doi: 10.3390/insects9030110 (PMC6163675; doi:10.3390/insects9030110)
Supplement: Supplementary file 1 [file insects-09-00110-s001.pdf]

insects

Supplementary Materials

Article

Nutritional Quality during Development Alters Insulin-Like Peptides' Expression and Physiology of the Adult Yellow Fever Mosquito, *Aedes aegypti*

Rana Pooraiiouby [1,†], Arvind Sharma [2,†], Joshua Beard [2], Jeremiah Reyes [2], Andrew Nuss [1,2,*] and Monika Gulia-Nuss [2,*]

1 Department of Agriculture, Nutrition, and Veterinary Sciences, University of Nevada, Reno, NV 89557, USA; pooraiiouby@googlemail.com
2 Department of Biochemistry and Molecular Biology, University of Nevada, Reno, NV 89557, USA; arvinds@unr.edu (A.S.); jdbeard93@gmail.com (J.B.); jeremiahbreyes@nevada.unr.edu (J.R.)
* Correspondence: nuss@cabnr.unr.edu (A.N.); mgulianuss@unr.edu (M.G.-N.); Tel.: +1-775-327-5096 (A.N.); +1-775-682-7333 (M.G.-N.)
† These authors contributed equally to this work.

Received: 05 June 2018; Accepted: 22 August 2018; Published: 30 August 2018

1. Methods

Nutrient Levels in Diets. To compare the carbohydrate and protein levels in PR and CR diets, we measured 150 mg of both diets equivalent to day-1 of the feeding schedule. Six replicates per diet were used. Levels were measured using Anthrone and Bradford assays for carbohydrates and proteins, respectively, as indicated in the Methods section of the main text (N = 6).

Life Span and Body Size. For life span, 40 adult males and females were kept together in small cages (clear gallon container). Mosquitoes were provided with water and 10% sugar solution. Mortality was recorded daily throughout the experiment. Dead / morbid adults were checked under the microscope and the morbid adults were returned to the cages and scored dead that did not respond to CO_2 and the legs were curled together. The experiment was replicated thrice with 40 mosquitoes of each sex from a different biological cohort during each replicate (n = 120).

For body size, wings from 15 males and females of each treatment were detached and mounted on a slide with mineral oil. A photograph was taken of each wing at 1.25× magnification using a camera mounted on a Leica M125 microscope. The microscope was calibrated with a ruler to standardize size measurements. Data were analyzed by unpaired t-test where male and female samples were analyzed separately.

2. Results

From the product labels, the PR diet was reported to contain ≥46% protein, ≥12% lipid, ≤3% carbohydrates; the CR diet ≤20% protein, ≥10% lipid and ≥20% carbohydrates. The percentage protein and carbohydrate we measured in each diet differed from these labels. However, the trend in imbalance between protein and carbohydrate components estimated from product labels remained. The PR diet contained approximately 50% protein, 15% carbohydrates, and the CR diet containing approximately 6% protein, 83% carbohydrate. The PR diet had approximately 7.5 times more protein than the CR diet. Similarly, CR diet had 5 times higher carbohydrate levels (Figure S1).

Adults eclosed from the PR diet fed larvae had longer lifespan. Females eclosed from a CR diet were more susceptible to dying earlier than males. 30% of the males on a PR diet survived longer than those on a CR diet, whereas this difference was 50% in females) (Figure S2).

There was no difference in body size in males whereas females of PR diet were significantly bigger than CR diet (Figure S3).

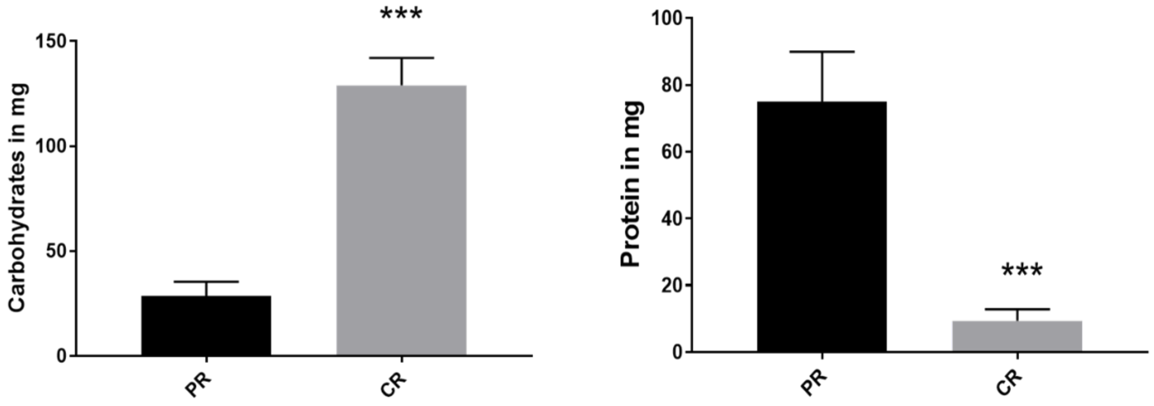

Figure S1. Levels of proteins and carbohydrates in different diets. 150 mg each of protein-rich (PR) and carbohydrate rich (CR) diets were used to determine proportions of these macronutrients. Samples were collected in six different tubes (N = 6) for each diet. Data were analyzed by unpaired *t*-test. *** $p < 0.001$.

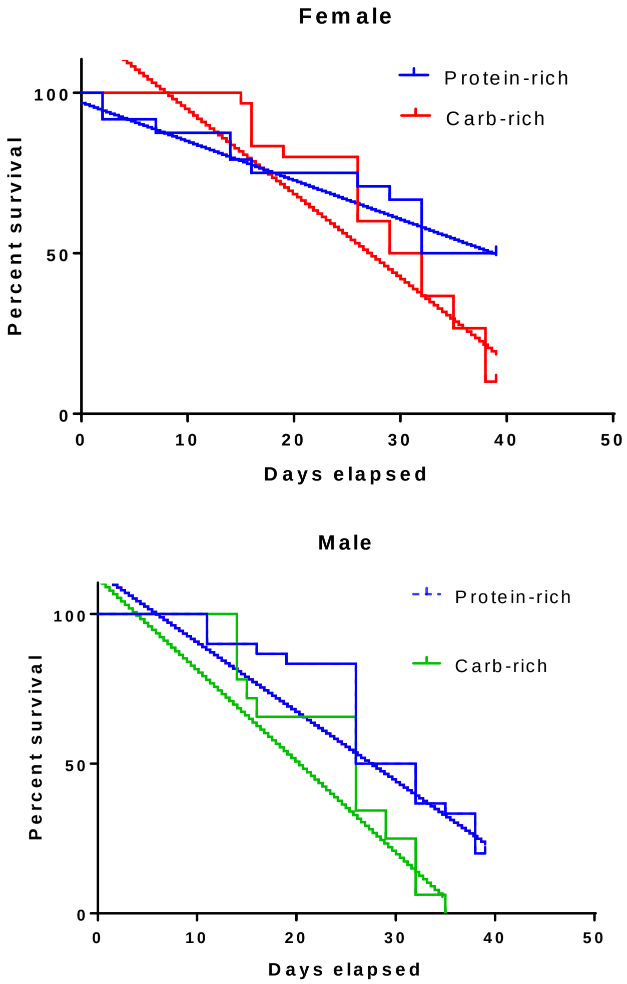

Figure S2. Effect of larval diets on adult mosquito survival. Forty males and females each were kept together in the cages made out of clear gallon containers. Cotton pads saturated with water and 10% sucrose were kept on the top of the cage. Pads were changed daily for continuous access to water and food to avoid desiccation. Mosquitoes were checked daily for 40 days for mortality and dead ones were removed from the cages. The number of mosquitoes dead in each group were plotted. Analysis was performed using Log-rank

(Mantel-Cox) test. Experiments were replicated thrice with different cohorts of mosquitoes. n = 120. Protein rich diet; Carbohydrate-rich diet, M= male; F= female. Female: $p = 0.02$; male: $p = 0.001$.

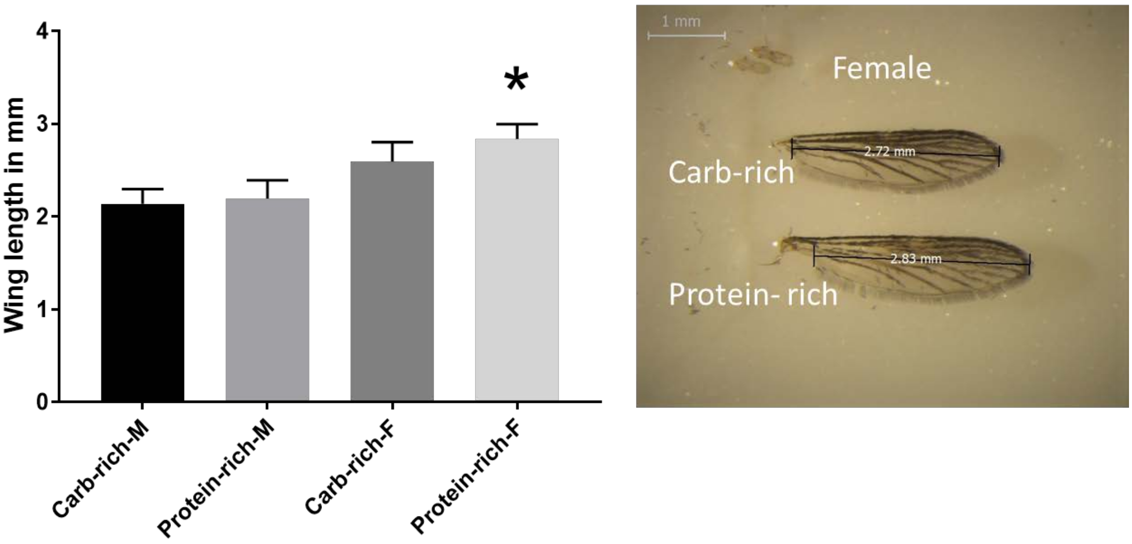

Figure S3. Effect of larval diets on adult mosquito body size**.** Wings from 15 males and females of each treatment were detached and mounted on a slide with mineral oil. Photographs were taken of each wing at 1.25x magnification using a camera mounted on a Leica M125 microscope. Data were analyzed by unpaired t-test where male and female samples were analyzed separately. A representative image of wings of females is shown. Female wings of the protein-rich diet were significantly longer than those reared on the carbohydrate-rich diet ($p = 0.02$). Male wings were of the same size in both treatments.

© 2018 by the authors. Submitted for possible open access publication under the terms and conditions of the Creative Commons Attribution (CC BY) license (http://creativecommons.org/licenses/by/4.0/).
